# Cardiovascular Autonomic Dysfunction and Falls in People With Multiple Sclerosis: Is There a Link? An Opinion Article

**DOI:** 10.3389/fnins.2020.610917

**Published:** 2020-12-07

**Authors:** Tobia Zanotto, Manuel E. Hernandez, Cristina N. Medrano, Kenneth R. Wilund, Jacob J. Sosnoff

**Affiliations:** ^1^Motor Control Research Laboratory, Department of Kinesiology and Community Health, University of Illinois at Urbana-Champaign, Urbana, IL, United States; ^2^Illinois Multiple Sclerosis Research Collaborative, University of Illinois at Urbana-Champaign, Urbana, IL, United States; ^3^Mobility and Fall Prevention Research Laboratory, Department of Kinesiology and Community Health, University of Illinois at Urbana-Champaign, Urbana, IL, United States; ^4^McKinley Health Center, University of Illinois at Urbana-Champaign, Urbana, IL, United States; ^5^Renal and Cardiovascular Disease Research Laboratory, Department of Kinesiology and Community Health, University of Illinois at Urbana-Champaign, Urbana, IL, United States

**Keywords:** dysautonomia, orthostatic hypotension, accidental falls, cardiovascular autonomic dysfunction, multiple sclerosis

## Introduction

Falls and fall-related injuries are highly prevalent in people living with multiple sclerosis (MS). A multinational meta-analysis revealed that 56% of people with MS (pwMS) fall at least once in any 3-month period (Nilsagård et al., 2015) and 11–42% of falls are injurious (Gunn et al., 2014; Mazumder et al., 2014). Falls are of clinical concern as they increase the burden of morbidity and mortality (Grossman et al., 2018). Mobility impairments, progressive MS, and cognitive impairment are the most commonly reported risk factors for falls in this clinical population (Gunn et al., 2013; Giannì et al., 2014; Sosnoff and Sung, 2015).

Despite the recent progress in disease-modifying medical treatments, as well as the numerous randomized controlled trials aiming to reduce falls, the evidence regarding effectiveness of interventions for fall-prevention in MS remains inconclusive (Hayes et al., 2019). Although such inconclusiveness is potentially related to the lack of methodological quality of intervention studies, it also highlights the need for accurate characterization of fall-etiology in this patient population. The success of fall-prevention programs is often based on their ability to effectively target underlying physiologic conditions. Therefore, in order to correctly prioritize rehabilitation strategies in MS, it is essential to identify all modifiable or partially-modifiable risk factors for falls.

Multiple sclerosis (MS) is characterized by damage to the central nervous system (CNS) as a result of inflammatory demyelination and neurodegeneration (Ghasemi et al., 2017). While such damage often translates into impairments of cognition and motor function, which are reasonably recognized as the main determinants of falls (Gunn et al., 2013), a growing body of research has explored the relationship between MS-related CNS damage and autonomic dysfunction (Racosta et al., 2015; Findling et al., 2020). Autonomic dysfunction can severely impact the quality of life in pwMS and often affects multiple organs and systems including the bladder, bowels, heart, as well as sexual and sudomotor functions (McDougall and McLeod, 2003; Lensch and Jost, 2011). A meta-analysis has concluded that cardiovascular autonomic dysfunction (CAD) is highly prevalent, with 19–42% of pwMS affected by this condition (Racosta et al., 2015). Importantly, CAD is one of the main drivers of orthostatic hypotension (OH) and contributes to symptoms of orthostatic intolerance, such as dizziness, which can directly or indirectly increase the risk of falling (Magkas et al., 2019). In this article, we aimed to outline the potential relationship between CAD and falls in pwMS.

## Cardiovascular Autonomic Dysfunction in MS

While pathophysiological processes of inflammation and neurodegeneration are thought to contribute to the development of CAD, the etiology of autonomic dysfunction in MS has not been elucidated (Findling et al., 2020). It has been postulated that CNS lesions, as assessed through magnetic resonance imaging, may be involved in the pathogenesis of CAD. For instance, several observational studies found that brainstem (Habek et al., 2016), midbrain (Saari et al., 2004), hippocampal (Winder et al., 2019), and spinal cord (de Seze et al., 2001) lesions were associated with indices of cardiovascular dysfunction including reduced heart rate variability (HRV) and increased blood pressure variability. However, due to conflicting evidence in the literature (Nasseri et al., 1999; Damla et al., 2018), it is not possible to make any conclusive statement as to whether CAD may be caused by CNS damage or by other epiphenomena of disrupted autonomic balance (Findling et al., 2020).

Common pharmacological treatments used in MS, such as immunotherapies, have also been linked to the development of CAD. Particularly, patients are often prescribed with Fingolimod, a sphingosine 1-phosphate receptor modulator (Chaudhry et al., 2017). Sustained usage of this immunomodulating drug has consistently been associated with impairments of cardiac autonomic function in pwMS. For instance, longitudinal studies have shown that prolonged treatment with Fingolimod significantly reduced both HRV and cardiovagal baroreflex sensitivity (BRS) (Racca et al., 2016; Simula et al., 2016; Vehoff et al., 2017). Additionally, administration of high dosage intravenous corticosteroids (e.g., methylprednisolone) and polypharmacy may also negatively affect cardiovascular autonomic function in MS (Vasheghani-Farahani et al., 2011; Findling et al., 2020).

The clinical implications of CAD in the context of MS are not clear. A large population-based cohort study revealed that, within 1 year of diagnosis, patients had an increased risk of stroke, myocardial infarction, and heart failure compared to the general population (Christiansen et al., 2010) and it was suggested that CAD may be involved in the etiological pathways of cardiovascular disease (Kaplan et al., 2015). Notably, the baroreflex plays a central role in buffering both the increases and decreases in blood pressures that normally occur in daily life (Kaufmann et al., 2020). Thus, baroreflex dysfunction represents a risk factor for both cardiovascular complications such as stroke and myocardial infarction (La Rovere et al., 2008; Lin et al., 2019), but also for conditions such as orthostatic dizziness, which is highly prevalent in pwMS (Habek et al., 2016).

## Relationship Between CAD and Falls

While there is growing evidence that CAD can manifest in OH or symptoms of orthostatic intolerance/dizziness due to baroreflex dysfunction in MS (Racosta et al., 2015), its relationship with falls has not received much scrutiny. Cross-sectional studies have consistently reported that, compared to age-matched healthy controls, pwMS have reduced HRV and baroreflex function (Tombul et al., 2011; Shirbani et al., 2018), both of which have been found to predict falls in patient populations characterized by dysautonomia (Castaldo et al., 2017; Razjouyan et al., 2017; Terroba-Chambi et al., 2020; Zanotto et al., 2020). A direct biological mechanism linking baroreflex dysfunction and falls can be reasonably theorized. Specifically, the impaired ability of baroreceptors to evoke tachycardia and vasoconstriction to effectively buffer a fall in blood pressure (BP) during an orthostatic challenge may lead to sudden onset of cerebral hypoperfusion (Kaufmann et al., 2020).

Orthostatic hypotension is a significant hypoperfusion-mediated risk factor for falls in the general geriatric population (Mol et al., 2019). In the context of MS, observational studies employing self-report or sphygmomanometer BP measurements have reported a high prevalence of OH or presyncope/syncope in middle-aged patients (36–38 years-old), ranging from 18–24% (Al-Araji et al., 2003; Kale et al., 2009). This is comparable to the prevalence (17.3%) found in much older populations (74 years-old) of community-dwelling adults (Cooke et al., 2013). Notably, even subtle alterations of BP response to orthostatic challenges (i.e., without a clear diagnosis of OH) can be predictive of falls (Shaw et al., 2015). Symptoms of orthostatic intolerance/postural dizziness are also highly prevalent (50–67%) in MS populations (both remitting-relapsing and progressive MS) (Flachenecker et al., 1999; Gunal et al., 2002; Al-Araji et al., 2003; Habek et al., 2016), which could be indicative of BP decrements during changes of body posture or prolonged standing.

Although falls caused by cerebral hypoperfusion are sometimes classified as syncopal events and therefore excluded from the operational definition of a fall, the etiology of falling is complex and often multifactorial (World Health Organization, 2008). Therefore, hypoperfusion symptoms such as dizziness may also act as secondary contributors to falls that are mainly precipitated by other factors such as sudden losses of balance or environmental hazards. International research collaboratives such as the ProFaNE group have recommended against excluding OH-related falls from the operational definition, as this would introduce a definitional artifact leading to biased findings (Lamb et al., 2005). Furthermore, the importance of recording fall-related circumstances has been emphasized (Hauer et al., 2006; Coote et al., 2014). In this regard, prospective cohort studies conducted in MS populations have often failed to report perceived causes of falling (Chinnadurai et al., 2018; Tajali et al., 2019), which limits the ability to identify precipitating factors. An observational study by Gunn et al. (2014) revealed that only 1.8% of recorded falls were directly attributed to dizziness/fainting, as opposed to a much greater proportion of falls that were precipitated by a loss of balance (19.4%) or legs giving way (8.7%). However, participants could not describe the perceived cause for the great majority of falls experienced (49%) in this study. While this observation is most likely related to the multifactorial etiology of falling and recall-bias, it could be hypothesized that CAD may represent a “hidden” contributing factor due to the fact that unexplained falls are often attributed to factors such as OH (Finucane et al., 2017; van Wijnen et al., 2017), which can be asymptomatic (Freeman et al., 2020).

Fatigue-related processes, in addition to cerebral hypoperfusion, may underscore the mechanisms by which CAD leads to falls in MS. Several studies have focused on the relationship between autonomic impairments of cardiac function and fatigability in pwMS (Lebre et al., 2007; Krbot Skorić et al., 2019). Lebre et al. (2007) observed that patients with fatigue had lower BP response to handgrip testing compared to those who were fatigue-free (14.6 ± 9.1 mmHg vs. 21.7 ± 7.2 mmHg, *p* < 0.05), postulating that impaired sympathetic outflow to the heart and vasculature may be a significant driver of fatigue. Similarly, Krbot Skorić et al. (2019) found negative correlations between Valsalva ratio and the modified fatigue impact scale (*r* = −0.306, *p* = 0.011) in a cohort with early-stage MS, suggesting an association between autonomic nervous system impairment and MS-related fatigue. Such evidence remains scarce and confounded by methodological design issues (e.g., a causative relationship cannot be inferred). However, a review by Sternberg (2017) has suggested that impaired neurovisceral integration of cardiovascular modulation, manifesting through the dysregulation of both sympathetic and parasympathetic responses, is involved in the etiology of multiple MS-related symptoms including fatigue. Additionally, CAD may contribute not only to physical but also cognitive fatigue in MS (Sander et al., 2017), as significant associations between cognitive decline and objective physiological markers of CAD such as reduced HRV (Sander et al., 2019) or blunted BP response to sustained handgrip testing (Niepel et al., 2013) have been highlighted. More importantly, prospective cohort studies of both healthy and neurological populations have suggested that OH arising from baroreflex and sympathetic dysfunction could lead to cognitive deterioration (e.g., through white matter lesions) as a result of chronic hypoperfusion and suboptimal cerebral autoregulation (Dadar et al., 2020; Zimmermann et al., 2020). Therefore, impairments of cognitive function, which are recognized as a risk factor for falls (Gunn et al., 2013; Sosnoff et al., 2013), may represent a further mediator of the relationship between CAD and fall-risk. It has been proposed that parasympathetic overactivity arising from inflammation-induced vagal activation would cause reductions in BP and orthostatic intolerance with concomitant impaired ability to maintain mental focus (Sander et al., 2017). In turn, cognitive impairment and/or fatigability can increase the risk of falling by impairing attention and the ability to evaluate intrinsic or extrinsic hazards during walking (Segev-Jacubovski et al., 2011).

Lastly, CAD seems to be more common among people affected by primary progressive MS (Findling et al., 2020), who tend to experience more falls compared to people with other subtypes of MS (Nilsagård et al., 2015). While this higher risk of falling is certainly related to greater overall disability (Penner et al., 2020), it is also plausible that autonomic impairments leading to orthostatic dysregulation of BP and/or fatigue may play a role in the higher occurrence of falls in progressive MS populations.

## Final Considerations

In this opinion article, we discussed the potential relationship between CAD and falls in people with MS. To the best of our knowledge, there is currently no information as to what proportion of patients suffering from falls is also affected by CAD. Although some preliminary evidence seems to indirectly point toward the existence of a mechanistic link between pathological alterations of cardiovascular autonomic function and falls in this clinical population, observational studies designed to explore this research hypothesis have yet to be conducted. Studies investigating purported physiological mechanisms of CAD, such as baroreflex dysfunction, in relation to signs and symptoms of orthostatic intolerance and falls would be a logical next step toward a better understanding of fall-etiology in MS. This research would have translational impact on rehabilitation strategies for fall-prevention if a significant relationship between CAD and falls was to emerge. Specifically, personalized pharmacologic/non-pharmacologic interventions targeting the underlying autonomic impairment may be considered as part of a more comprehensive fall-prevention strategy.

## Author Contributions

TZ drafted the original manuscript. MH, CM, KW, and JS critically revised the manuscript. All authors agree to be accountable for the content of the work.

## Conflict of Interest

The authors declare that the research was conducted in the absence of any commercial or financial relationships that could be construed as a potential conflict of interest.

## Abbreviations

BP: blood pressure
BRS: baroreflex sensitivity
CAD: cardiovascular autonomic dysfunction
CNS: central nervous system
HR: heart rate
HRV: heart rate variability
OH: orthostatic hypotension
MS: multiple sclerosis
pwMS: people with multiple sclerosis.

## References

[B1] Al-Araji A. H. Al-Mahdawi A. M. Mohammad A. I. (2003). Autonomic dysfunction in multiple sclerosis. Neurosciences 8, 177–183. 23649115

[B2] Castaldo R. Melillo P. Izzo R. De Luca N. Pecchia L. (2017). Fall prediction in hypertensive patients via short-term HRV analysis. IEEE J. Biomed. Health Inform. 21, 399–406. 10.1109/JBHI.2016.2543960 28113874

[B3] Chaudhry B. Z. Cohen J. A. Conway D. S. (2017). Sphingosine 1-Phosphate receptor modulators for the treatment of multiple sclerosis. Neurotherapeutics 14, 859–873. 10.1007/s13311-017-0565-4 28812220 PMC5722770

[B4] Chinnadurai S. A. Gandhirajan D. Srinivasan A. V. Kesavamurthy B. Ranganathan L. N. Pamidimukkala V. (2018). Predicting falls in multiple sclerosis: do electrophysiological measures have a better predictive accuracy compared to clinical measures? Mult. Scler. Relat. Disord. 20, 199–203. 10.1016/j.msard.2018.01.027 29414299

[B5] Christiansen C. F. Christensen S. Farkas D. K. Miret M. Sørensen H. T. Pedersen L. (2010). Risk of arterial cardiovascular diseases in patients with multiple sclerosis: a population-based cohort study. Neuroepidemiology 35, 267–274. 10.1159/000320245 20881430

[B6] Cooke J. Carew S. Quinn C. O'Connor M. Curtin J. O'Connor C.. (2013). The prevalence and pathological correlates of orthostatic hypotension and its subtypes when measured using beat-to-beat technology in a sample of older adults living in the community. Age Ageing 42, 709–714. 10.1093/ageing/aft112 23934598

[B7] Coote S. Sosnoff J. J. Gunn H. (2014). Fall incidence as the primary outcome in multiple sclerosis falls-prevention trials: recommendation from the international ms falls prevention research network. Int. J. MS Care 16, 178–184. 10.7224/1537-2073.2014-059 25694776 PMC4321456

[B8] Dadar M. Fereshtehnejad S. M. Zeighami Y. Dagher A. Postuma R. B. Collins D. L. (2020). White matter hyperintensities mediate impact of dysautonomia on cognition in Parkinson's disease. Mov. Disord. Clin. Pract. 7, 639–647. 10.1002/mdc3.13003 32775509 PMC7396867

[B9] Damla O. Altug C. Pinar K. K. Alper K. Dilek I. G. Kadriye A. (2018). Heart rate variability analysis in patients with multiple sclerosis. Mult. Scler. Relat. Disord. 24, 64–68. 10.1016/j.msard.2018.06.012 29957350

[B10] de Seze J. Stojkovic T. Gauvrit J. Y. Devos D. Ayachi M. Cassim F.. (2001). Autonomic dysfunction in multiple sclerosis: cervical spinal cord atrophy correlates. J. Neurol. 248, 297–303. 10.1007/s004150170204 11374094

[B11] Findling O. Hauer L. Pezawas T. Rommer P. S. Struhal W. Sellner J. (2020). Cardiac autonomic dysfunction in multiple sclerosis: a systematic review of current knowledge and impact of immunotherapies. J. Clin. Med. 9:335. 10.3390/jcm9020335 31991711 PMC7073977

[B12] Finucane C. O'Connell M. D. Donoghue O. Richardson K. Savva G. M. Kenny R. A. (2017). Impaired orthostatic blood pressure recovery is associated with unexplained and injurious falls. J. Am. Geriatr. Soc. 65, 474–482. 10.1111/jgs.14563 28295143

[B13] Flachenecker P. Wolf A. Krauser M. Hartung H. P. Reiners K. (1999). Cardiovascular autonomic dysfunction in multiple sclerosis: correlation with orthostatic intolerance. J. Neurol. 246, 578–586. 10.1007/s004150050407 10463360

[B14] Freeman R. Illigens B. M. W. Lapusca R. Campagnolo M. Abuzinadah A. R. Bonyhay I.. (2020). Symptom recognition is impaired in patients with orthostatic hypotension. Hypertension 75, 1325–1332. 10.1161/HYPERTENSIONAHA.119.13619 32223377 PMC12818701

[B15] Ghasemi N. Razavi S. Nikzad E. (2017). Multiple sclerosis: pathogenesis, symptoms, diagnoses and cell-based therapy. Cell J. 19, 1–10. 10.22074/cellj.2016.4867 28367411 PMC5241505

[B16] Giannì C. Prosperini L. Jonsdottir J. Cattaneo D. (2014). A systematic review of factors associated with accidental falls in people with multiple sclerosis: a meta-analytic approach. Clin. Rehabil. 28, 704–716. 10.1177/0269215513517575 24569653

[B17] Grossman D. C. Curry S. J. Owens D. K. Barry M. J. Caughey A. B. Davidson K. W.. (2018). Interventions to prevent falls in community-dwelling older adults: US preventive services task force recommendation statement. JAMA 319, 1696–1704. 10.1001/jama.2018.3097 29710141

[B18] Gunal D. I. Afsar N. Tanridag T. Aktan S. (2002). Autonomic dysfunction in multiple sclerosis: correlation with disease-related parameters. Eur. Neurol. 48, 1–5. 10.1159/000064949 12138302

[B19] Gunn H. Creanor S. Haas B. Marsden J. Freeman J. (2014). Frequency, characteristics, and consequences of falls in multiple sclerosis: findings from a cohort study. Arch. Phys. Med. Rehabil. 95, 538–545. 10.1016/j.apmr.2013.08.244 24055784

[B20] Gunn H. J. Newell P. Haas B. Marsden J. F. Freeman J. A. (2013). Identification of risk factors for falls in multiple sclerosis: a systematic review and meta-analysis. Phys. Ther. 93, 504–513. 10.2522/ptj.20120231 23237970

[B21] Habek M. Crnošija L. Lovrić M. Junaković A. Krbot Skorić M. Adamec I. (2016). Sympathetic cardiovascular and sudomotor functions are frequently affected in early multiple sclerosis. Clin. Auton. Res. 26, 385–393. 10.1007/s10286-016-0370-x 27448576

[B22] Hauer K. Lamb S. E. Jorstad E. C. Todd C. Becker C. PROFANE-Group (2006). Systematic review of definitions and methods of measuring falls in randomised controlled fall prevention trials. Age Ageing 35, 5–10. 10.1093/ageing/afi218 16364930

[B23] Hayes S. Galvin R. Kennedy C. Finlayson M. McGuigan C. Walsh C. D.. (2019). Interventions for preventing falls in people with multiple sclerosis. Cochrane Database Syst Rev. 11:CD012475. 10.1002/14651858.CD012475.pub2 31778221 PMC6953359

[B24] Kale N. Magana S. Agaoglu J. Tanik O. (2009). Assessment of autonomic nervous system dysfunction in multiple sclerosis and association with clinical disability. Neurol. Int. 1:e5. 10.4081/ni.2009.e4 21577363 PMC3093232

[B25] Kaplan T. B. Berkowitz A. L. Samuels M. A. (2015). Cardiovascular dysfunction in multiple sclerosis. Neurologist 20, 108–114. 10.1097/NRL.0000000000000064 26671744

[B26] Kaufmann H. Norcliffe-Kaufmann L. Palma J. A. (2020). Baroreflex dysfunction. N. Engl. J. Med. 382, 163–178. 10.1056/NEJMra1509723 31914243

[B27] Krbot Skorić M. Crnošija L. Adamec I. Barun B. Gabelić T. Smoljo T.. (2019). Autonomic symptom burden is an independent contributor to multiple sclerosis related fatigue. Clin. Auton. Res. 29, 321–328. 10.1007/s10286-018-0563-6 30209702

[B28] La Rovere M. T. Pinna G. D. Raczak G. (2008). Baroreflex sensitivity: measurement and clinical implications. Ann. Noninvasive Electrocardiol. 13, 191–207. 10.1111/j.1542-474X.2008.00219.x 18426445 PMC6931942

[B29] Lamb S. E. Jørstad-Stein E. C. Hauer K. Becker C. Prevention of Falls Network Europe and Outcomes Consensus Group (2005). Development of a common outcome data set for fall injury prevention trials: the prevention of falls network europe consensus. J. Am. Geriatr. Soc. 53, 1618–1622. 10.1111/j.1532-5415.2005.53455.x 16137297

[B30] Lebre A. T. Mendes M. F. Tilbery C. P. Almeida A. L. Scatolini Neto A. (2007). Relação entre fadiga e distúrbios autonômicos na esclerose múltipla [Relation between fatigue and autonomic disturbances in multiple sclerosis]. Arq. Neuropsiquiatr. 65, 663–668. 10.1590/S0004-282X2007000400023 17876411

[B31] Lensch E. Jost W. H. (2011). Autonomic disorders in multiple sclerosis. Autoimmune Dis. 2011:803841. 10.4061/2011/803841 21603189 PMC3096149

[B32] Lin C. H. Yen C. C. Hsu Y. T. Chen H. H. Cheng P. W. Tseng C. J.. (2019). Baroreceptor sensitivity predicts functional outcome and complications after acute ischemic stroke. J. Clin. Med. 8:300. 10.3390/jcm8030300 30832391 PMC6462921

[B33] Magkas N. Tsioufis C. Thomopoulos C. Dilaveris P. Georgiopoulos G. Sanidas E. (2019). Orthostatic hypotension: from pathophysiology to clinical applications and therapeutic considerations. J. Clin. Hypertens. 21, 546–554. 10.1111/jch.13521 PMC8030387 30900378

[B34] Mazumder R. Murchison C. Bourdette D. Cameron M. (2014). Falls in people with multiple sclerosis compared with falls in healthy controls. PLoS ONE 9:e107620. 10.1371/journal.pone.0107620 25254633 PMC4177842

[B35] McDougall A. J. McLeod J. G. (2003). Autonomic nervous system function in multiple sclerosis. J. Neurol. Sci. 215, 79–85. 10.1016/S0022-510X(03)00205-3 14568133

[B36] Mol A. Bui Hoang P. T. S. Sharmin S. Reijnierse E. M. van Wezel R. J. A. Meskers C. G. M.. (2019). Orthostatic hypotension and falls in older adults: a systematic review and meta-analysis. J. Am. Med. Dir. Assoc. 20, 589–597. 10.1016/j.jamda.2018.11.003 30583909

[B37] Nasseri K. Uitdehaag B. M. van Walderveen M. A. Ader H. J. Polman C. H. (1999). Cardiovascular autonomic function in patients with relapsing remitting multiple sclerosis: a new surrogate marker of disease evolution? Eur. J. Neurol. 6, 29–33. 10.1046/j.1468-1331.1999.610029.x 10209346

[B38] Niepel G. Bibani R. H. Vilisaar J. Langley R. W. Bradshaw C. M. Szabadi E. (2013). Association of a deficit of arousal with fatigue in multiple sclerosis: effect of modafinil. Neuropharmacology 64, 380–388. 10.1016/j.neuropharm.2012.06.036 22766394

[B39] Nilsagård Y. Gunn H. Freeman J. Hoang P. Lord S. Mazumder R.. (2015). Falls in people with MS–an individual data meta-analysis from studies from Australia, Sweden, United Kingdom and the United States. Mult. Scler. 21, 92–100. 10.1177/1352458514538884 24948687 PMC4361466

[B40] Penner I. K. McDougall F. Brown T. M. Slota C. Doward L. Julian L.. (2020). Exploring the impact of fatigue in progressive multiple sclerosis: a mixed-methods analysis. Mult. Scler. Relat. Disord. 43:102207. 10.1016/j.msard.2020.102207 32505026

[B41] Racca V. Rovaris M. Vaini E. Cavarretta R. Ferratini M. Toccafondi A.. (2016). 6-month effects of fingolimod on indexes of cardiovascular autonomic control in multiple sclerosis. J. Am. Coll. Cardiol. 68, 2027-2029. 10.1016/j.jacc.2016.08.032 27788858

[B42] Racosta J. M. Sposato L. A. Morrow S. A. Cipriano L. Kimpinski K. Kremenchutzky M. (2015). Cardiovascular autonomic dysfunction in multiple sclerosis: a meta-analysis. Mult. Scler. Relat. Disord. 4, 104–111. 10.1016/j.msard.2015.02.002 25787186

[B43] Razjouyan J. Grewal G. S. Rishel C. Parthasarathy S. Mohler J. Najafi B. (2017). Activity monitoring and heart rate variability as indicators of fall risk: proof-of-concept for application of wearable sensors in the acute care setting. J. Gerontol. Nurs. 43, 53–62. 10.3928/00989134-20170223-01 28253410

[B44] Saari A. Tolonen U. Pääkkö E. Suominen K. Pyhtinen J. Sotaniemi K.. (2004). Cardiovascular autonomic dysfunction correlates with brain MRI lesion load in MS. Clin. Neurophysiol. 115, 1473–1478. 10.1016/j.clinph.2004.01.012 15134718

[B45] Sander C. Hildebrandt H. Schlake H. P. Eling P. Hanken K. (2017). Subjective cognitive fatigue and autonomic abnormalities in multiple sclerosis patients. Front. Neurol. 8:475. 10.3389/fneur.2017.00475 28955298 PMC5601401

[B46] Sander C. Modes F. Schlake H. P. Eling P. Hildebrandt H. (2019). Capturing fatigue parameters: the impact of vagal processing in multiple sclerosis related cognitive fatigue. Mult. Scler. Relat. Disord. 32, 13–18. 10.1016/j.msard.2019.04.013 31005825

[B47] Segev-Jacubovski O. Herman T. Yogev-Seligmann G. Mirelman A. Giladi N. Hausdorff J. M. (2011). The interplay between gait, falls and cognition: can cognitive therapy reduce fall risk? Expert Rev. Neurother. 11, 1057–1075. 10.1586/ern.11.69 21721921 PMC3163836

[B48] Shaw B. H. Loughin T. M. Robinovitch S. N. Claydon V. E. (2015). Cardiovascular responses to orthostasis and their association with falls in older adults. BMC Geriatr. 15:174. 10.1186/s12877-015-0168-z 26703012 PMC4690276

[B49] Shirbani F. Barin E. Lee Y. C. Ng K. Parratt J. D. E. Butlin M.. (2018). Characterisation of cardiac autonomic function in multiple sclerosis based on spontaneous changes of heart rate and blood pressure. Mult. Scler. Relat. Disord. 22, 120–127. 10.1016/j.msard.2018.03.018 29656272

[B50] Simula S. Laitinen T. Laitinen T. M. Tarkiainen T. Hartikainen P. Hartikainen J. E. (2016). Effect of fingolimod on cardiac autonomic regulation in patients with multiple sclerosis. Mult. Scler. 22, 1080–1085. 10.1177/1352458515604384 26362903

[B51] Sosnoff J. J. Balantrapu S. Pilutti L. A. Sandroff B. M. Morrison S. Motl R. W. (2013). Cognitive processing speed is related to fall frequency in older adults with multiple sclerosis. Arch. Phys. Med. Rehabil. 94, 1567–1572. 10.1016/j.apmr.2013.02.009 23422407

[B52] Sosnoff J. J. Sung J. (2015). Reducing falls and improving mobility in multiple sclerosis. Expert Rev. Neurother. 15, 655–666. 10.1586/14737175.2015.1046377 25973774

[B53] Sternberg Z. (2017). Impaired neurovisceral integration of cardiovascular modulation contributes to multiple sclerosis morbidities. Mol. Neurobiol. 54, 362–374. 10.1007/s12035-015-9599-y 26742516

[B54] Tajali S. Mehravar M. Negahban H. van Dieën J. H. Shaterzadeh-Yazdi M. J. Mofateh R. (2019). Impaired local dynamic stability during treadmill walking predicts future falls in patients with multiple sclerosis: a prospective cohort study. Clin. Biomech. 67, 197–201. 10.1016/j.clinbiomech.2019.05.013 31234121

[B55] Terroba-Chambi C. Bruno V. Vigo D. E. Merello M. (2020). Heart rate variability and falls in Huntington's disease. Clin. Auton. Res. 10.1007/s10286-020-00669-2. [Epub ahead of print]. 32026136

[B56] Tombul T. Anlar O. Tuncer M. Huseyinoglu N. Eryonucu B. (2011). Impaired heart rate variability as a marker of cardiovascular autonomic dysfunction in multiple sclerosis. Acta Neurol. Belg. 111, 116–120. 21748930

[B57] van Wijnen V. K. Finucane C. Harms M. P. M. Nolan H. Freeman R. L. Westerhof B. E.. (2017). Noninvasive beat-to-beat finger arterial pressure monitoring during orthostasis: a comprehensive review of normal and abnormal responses at different ages. J. Intern Med. 282, 468–483. 10.1111/joim.12636 28564488

[B58] Vasheghani-Farahani A. Sahraian M. A. Darabi L. Aghsaie A. Minagar A. (2011). Incidence of various cardiac arrhythmias and conduction disturbances due to high dose intravenous methylprednisolone in patients with multiple sclerosis. J. Neurol. Sci. 309, 75–78. 10.1016/j.jns.2011.07.018 21831398

[B59] Vehoff J. Haegele-Link S. Humm A. Kaegi G. Mueller S. K. Sauter R.. (2017). Heart rate variability decreases after 3 months of sustained treatment with fingolimod. J. Neurol. 264, 2313–2317. 10.1007/s00415-017-8636-3 28993873

[B60] Winder K. Linker R. A. Seifert F. Wang R. Lee D. H. Engelhorn T.. (2019). Cerebral lesion correlates of sympathetic cardiovascular activation in multiple sclerosis. Hum. Brain Mapp. 40, 5083–5093. 10.1002/hbm.24759 31403742 PMC6865522

[B61] World Health Organization (2008). Global Report on Falls Prevention in Older Age (W.H.O. Ageing, L. C. Unit).

[B62] Zanotto T. Mercer T. H. van der Linden M. L. Rush R. Traynor J. P. Petrie C. J.. (2020). The relative importance of frailty, physical and cardiovascular function as exercise-modifiable predictors of falls in haemodialysis patients: a prospective cohort study. BMC Nephrol. 21:99. 10.1186/s12882-020-01759-z 32169050 PMC7071740

[B63] Zimmermann M. Wurster I. Lerche S. Roeben B. Machetanz G. Sünkel U.. (2020). Orthostatic hypotension as a risk factor for longitudinal deterioration of cognitive function in the elderly. Eur. J. Neurol. 27, 160–167. 10.1111/ene.14050 31342593

